# Small Fibre Involvement in Multifocal Motor Neuropathy Explored with Sudoscan: A Single-Centre Experience

**DOI:** 10.3390/diagnostics10100755

**Published:** 2020-09-26

**Authors:** Marco Luigetti, Silvia Giovannini, Angela Romano, Giulia Bisogni, Francesco Barbato, Andrea Di Paolantonio, Serenella Servidei, Giuseppe Granata, Mario Sabatelli

**Affiliations:** 1 Fondazione Policlinico Universitario A. Gemelli IRCCS, UOC Neurologia, 00168 Rome, Italy; 2 Università Cattolica del Sacro Cuore, 00168 Rome, Italy; silvia.giovannini@policlinicogemelli.it (S.G.); angela.romano12@gmail.com (A.R.); francescobarbato.88@hotmail.it (F.B.); andrea.dp1988@gmail.com (A.D.P.); serenella.servidei@unicatt.it (S.S.); granata.gius@gmail.com (G.G.); mario.sabatelli@unicatt.it (M.S.); 3 Fondazione Policlinico Universitario A. Gemelli IRCCS, UOC Riabilitazione, 00168 Rome, Italy; 4 Centro Clinico NEMO adulti, 00168 Rome, Italy; giulia.bisogni@centrocliniconemo.it; 5 Fondazione Policlinico Universitario A. Gemelli IRCCS, UOC Neurofisiopatologia, 00168 Rome, Italy

**Keywords:** polyneuropathy, sensory involvement, sudoscan, small fibres, multifocal motor neuropathy

## Abstract

Objective: Multifocal motor neuropathy (MMN) is a rare inflammatory neuropathy, clinically characterized by exclusive motor involvement. We wished to evaluate the possible presence of sensory dysfunction, including the evaluation of small fibres, after a long-term disease course. Patients and methods: seven MMN patients, regularly followed in our Neurology Department, underwent clinical evaluation, neurophysiological examination by nerve conduction studies (NCSs), and Sudoscan. We compared neurophysiological data with a group of patients with other disorders of the peripheral nervous system. Results: NCSs showed a reduction of sensory nerve action potential amplitude in 2/7 MMN patients. Sudoscan showed borderline electrochemical skin conductance (ESC) values in 3/7 MMN patients (two of them with abnormal sensory NCSs). Conclusions: Our results confirm that sensory involvement may be found in some MMN after a long-term disease course, and it could also involve the small fibres.

## 1. Introduction

MMN (multifocal motor neuropathy) is a rare disorder in which focal areas of multiple motor nerves are attacked by one’s own immune system [1,2,3]. Antibodies to ganglioside GM1 are reported in 40–85% of cases [4,5,6,7]. Typically, MMN is a slowly progressive disorder, resulting in asymmetric limb weakness; patients frequently develop weakness in their hand(s), resulting in dropping of objects or sometimes inability to turn a key in a lock. The weakness associated with MMN can be recognized as fitting a specific nerve territory. There is essentially no numbness, tingling, or pain [1,2,3,4,5,6,7]. Classically, nerve conduction studies reveal normal sensory neurography and the presence of conduction blocks (CBs) without slowing of motor nerve conduction velocities [1,2,3,4,5,6,7]. Corticosteroids and plasma exchange are not effective, while treatment with intravenous immunoglobulin (IVIg) and/or cyclophosphamide generally delays or stops disease progression [8,9,10,11].

Even if sensory involvement is typically absent, it can be subclinical after a long-term disease course [3,12,13,14,15]. Indeed, sural nerve biopsies have shown minimal changes, perhaps suggestive of demyelination in some cases [16], and some sensory symptoms, including neuropathic pain, have been sometimes reported by patients [17,18], the latter being commonly associated with damage of small fibre involvement. Despite these evidences, small fibres have never been specifically investigated in patients.

Sudoscan is a fairly recent technique that provides a quick, non-invasive and quantitative assessment of the sudomotor function [19]. It combines low direct current stimulation and reverse iontophoresis as a way of measuring the local conductance derived from the electrochemical reaction between the sweat chloride and the nickel electrodes [16]. At these low voltages, the stratum corneum acts as a capacitor, making the measured current only dependent on the chloride production by the sweat glands. The electrochemical skin conductance (ESC) is then expressed in microSiemens (μS) [19]. Sudomotor dysfunction is one of the earliest detectable abnormalities in distal small fibre neuropathies, considering that sweat glands are innervated by sudomotor, postganglionic, thin, unmyelinated cholinergic sympathetic C-fibres, and a number of skin biopsy studies have shown a reduction in the epidermal C-nerve fibres in patients with diabetes [19]. Indeed, Sudoscan has been recently described as a promising tool in the assessment of sudomotor dysfunction in diabetic small fibre neuropathy [19,20], in mitochondrial diseases [21], and in amyloid neuropathy [22].

For this purpose, in this study, we examined a cohort of MMN patients, regularly followed in our department, in order to investigate the possible presence of small fibre dysfunction after a long-term disease course by the use of Sudoscan.

## 2. Materials and Methods

### 2.1. Patients

We examined seven MMN adult patients regularly followed at our Neurology Department. All patients fulfilled the clinical and neurophysiological diagnostic criteria for MMN at initial examination [23]. Besides a complete neurological examination and neurophysiological assessment, all patients underwent extensive laboratory screening to rule out other possible causes of neuropathy (fasting plasma glucose, glycosylated hemoglobin, fT3, fT4, TSH, anti-thyroid antibodies, serum vitamin B12 and folates, hepatic enzymes, creatinine, urinalysis, antinuclear antibodies, anti-extractible nuclear antigens antibodies, anti-DNA antibodies, anti-neutrophil cytoplasmic antibodies, circulating C3 and C4, screening for celiac disease, alcohol use, and serologic tests for HBV, HCV and HIV). All lab tests were repeated at the time of the last neurophysiological examination in order to detect a possible occurrence of a further cause of sensory impairment. Currently, all patients are regularly treated with monthly cycles of high dose IVIg. The presence of pain and/or autonomic symptoms (i.e., diarrhea; alternation of constipation and diarrhea; dry eye or mouth; urinary incontinence or retention; sexual disturbances) was investigated with targeted questions. Assessment for orthostatic hypotension was also carried out. Pain, if present, was scored with the Numeric Rating Scale (NRS).

A group of patients with different disorders involving the peripheral nervous system was used as control, including seven patients affected by definite amyotrophic lateral sclerosis (ALS) according to revised El Escorial criteria [24] and four patients with chronic inflammatory demyelinating neuropathy according to European Federation of Neurological Societies and Peripheral Nerve Society (EFNS/PNS) criteria [25].

### 2.2. Neurophysiological Tests

MMN was defined at diagnosis according to EFNS/PNS neurophysiological criteria [23]. The nerve conduction study (NCS) technique was explained in detail in previous papers [26,27,28,29]. We examined median and ulnar nerves bilaterally in upper limbs, and peroneal, tibial and sural nerves in the lower limb contralateral to the most affected hand at the initial examination and at last follow-up. For motor nerves, we considered as abnormal a compound muscle action potential (CMAP) having an amplitude <5 mV, while for sensory nerves we considered as pathological an amplitude of sensory nerve action potential (SNAP) <5 μV; these normative data were obtained after control analysis was performed in our neurophysiology laboratory [27,28,29].

Sudoscan was performed asking the patients to put their hands and feet on the plate electrodes and to stand still for 3 min. A mean score of electrochemical skin conductance (ESC) for both hands and both feet was automatically calculated and analyzed by the machine [20]. An ESC ≥ 70 μS was considered normal; values between 53 and 69 μS were considered borderline; an ESC ≤ 52 μS was considered definitely abnormal [30]. The test was repeated twice ten minutes apart to guarantee consistency.

### 2.3. Statistical Analysis

Statistical analysis of data was performed by SPSS (Statistical Package for Social Science Statistics for Windows, Version 24.0. IBM Corp.: Armonk, NY, USA) to assess any differences between the group of patients. The Mann–Whitney *U* test and Fisher’s two-tailed exact test were used to compare numerical and nominal dichotomous variables, respectively. In case of categorical polytomous variables, a Chi-squared test was performed. Significance was set at 0.05.

### 2.4. Ethics

The study was approved by the Agostino Gemelli University Hospital Foundation IRCCS-Catholic University of the Sacred Heart Ethics Committee, Rome (Prot. 6464/19 (12309/19) ID2434, 16 October 2019). The study protocol conforms to the ethical guidelines of the 1975 Declaration of Helsinki (6th revision, 2008) as reflected in a priori approval by the Institution’s Human Research Committee. Written informed consent was obtained from all the participants.

## 3. Results

### 3.1. MMN Patients

Main demographic, clinical and neurophysiological data of the MMN patients are summarized in Table 1.

The seven enrolled MMN patients (five men and two women) had a mean age at onset of 31.7 years (median 35.0; standard deviation 11.2). Mean age at examination was 37.0 years (median 38.0; standard deviation 10.8). Mean follow-up from diagnosis was 51.4 months (median 36.0; standard deviation 24.3). Mean disease duration from symptom onset was 66.6 months (median 48.0; standard deviation 29.7). Neurological examination revealed muscle weakness in upper limbs involving one hand or both (Table 1) in all patients; extensor digitorum communis and extensor carpi (supplied by radial nerve) were also frequently affected, being involved in 5/7 patients (4/5 bilaterally). Sensory symptoms were reported only by one patient, namely paraesthesias in the most affected hand (Table 1). No patient reported pain or symptoms suggestive of autonomic involvement. Tendon reflexes were always present. IgM anti-GM1 antibodies, tested with ELISA, were positive in 4/7 (57%) patients (normal value 0–50 index; range in our patients 21–112; mean value 65.1; median value 64; standard deviation 32.8).

### 3.2. Neurophysiological Evaluation

NCSs at initial diagnosis confirmed MMN EFNS/PNS neurophysiological criteria [23]: in all patients, sensory nerve conduction studies were unremarkable, and CBs in motor nerves were present. NCSs at last follow-up showed a reduction of CMAP amplitude recorded from hand muscles in 5/7 patients (71%); in 3/7 (43%) the involvement of upper limbs was bilateral. In one patient, a reduction of peroneal CMAP amplitude in lower limbs was also observed. Conversely, a reduction of SNAP amplitude in upper limb nerves was observed in 2/7 patients (29%). SNAP amplitude reduction was always associated with CMAP amplitude reduction, involving the ulnar nerve bilaterally. NCSs did not detect a compression of the median nerve at wrist or ulnar nerve at the elbow, thus excluding a carpal tunnel syndrome or ulnar neuropathy at elbow. Furthermore, in patients with sensory involvement of the ulnar nerve, we also performed a nerve ultrasound at the elbow, with unremarkable results. CBs were not detected at last follow-up (on average 66.9 months), likely for the current therapy with IVIg and the long-lasting disease course. In the only patient who reported sensory symptoms, we observed a reduction of ulnar SNAP amplitude bilaterally. We performed electromyography in all muscles with reduced CMAP amplitude and we always confirmed the presence of fibrillation potentials and/or positive sharp waves suggestive of an axonal loss.

Sudoscan was normal in 4/7 patients (57%) and showed a borderline ESC value in the upper limbs in 3/7 patients (43%). An asymmetry between upper or lower limbs (considered if >10%) was never observed.

### 3.3. Control Group

As control groups, we included four CIDP and seven ALS patients. Main demographic, clinical and neurophysiological data of the control groups are summarized in Table 1.

Considering nerve conduction studies in ALS patients, we did not find any abnormalities of sensory nerve conduction studies or ESC explored with Sudoscan. In all of the motor nerves tested, CMAP was not detectable or showed reduced amplitude as expected.

All CIDP patients were chronic progressive forms in which the diagnosis was initially formulated according to EFNS/PNS criteria [19], and currently treated with regular infusions of IVIg. NCSs at last follow-up showed a reduction of CMAP amplitude recorded from hand muscles in 3/4 patients (75%); in 2/4 (50%) the involvement of upper limbs was bilateral. In one patient, a reduction of peroneal CMAP amplitude in the lower limbs was also observed. In all of these patients, an involvement of sensory fibres at the NCS was also found. Sudoscan was normal in 2/4 patients (50%), while it showed a borderline ESC value in 1/4 patients (25%), and a definitively abnormal value in the upper limbs in another one (1/4, 25%). An asymmetry between upper or lower limbs was never observed.

Comparing CIDP or ALS controls with MMN patients, no differences were found in gender distribution. Conversely, the mean age at onset for MMN patients was younger if compared to CIPD or ALS patients.

Regarding NCSs, CMAP amplitude was lower in ALS patients (but not in CIDP) if compared to MMN, while SNAP amplitude was lower in both ALS and CIDP patients if compared to MMN, with the only exception of ulnar nerve SNAP for ALS. Conversely, we did not find any difference between MMN patients and controls in ESC mean values.

Detailed statistical comparison between MMN patients and both CIDP and ALS controls is summarized in Table 2.

## 4. Discussion

Sensory disturbances classically rule out a clinical diagnosis of MMN, however many papers have reported sensory symptoms or subclinical sensory neurophysiological involvement in MMN patients [12,13,14,15,16,17,18]. While sensory symptoms and/or classical NCSs have been widely investigated, the impairment of small fibres has never been studied in MMN.

We examined our small cohort of MMN patients with Sudoscan, a new device largely used to test small fibre function in diabetes [19,20,31] and in different neuromuscular diseases, namely amyloid neuropathy [22,30,32] or mitochondrial diseases [33].

Our data confirmed a slight sensory involvement in our cohort of patients with MMN. Indeed, after a long disease course, two out of seven patients showed sensory abnormalities at the NCS; both patients also showed borderline ESC values, and, in one case, sensory symptoms were reported.

Interestingly, in both patients, sensory abnormalities were confined to upper limbs that are more frequently affected in MMN, and involved severely damaged nerves as demonstrated by the CMAP amplitude. We can speculate that, after long follow-up, small fibres can also suffer in this setting.

Furthermore, in another patient we found borderline ESC values with a normal NCS; longitudinal follow-up of this patient will clarify if an involvement of large sensory fibres will also appear.

As a control group, we included ALS patients, in which we did not find any sensory abnormalities, and CIDP patients, in which we found sensory abnormalities involving large fibres, small fibres or both, according to a possible focal distribution of the inflammatory process [34].

Comparing NCS results we found a lower CMAP amplitude in ALS patients with respect to MMN, probably caused by the severity of denervation characteristic of this disease. Conversely, regarding sensory NCSs, we generally found lower SNAP amplitude in both ALS and CIDP patients if compared with MMN; this data is easy to explain considering that sensory involvement is typical of CIDP, that ALS patients were older than MMN and that SNAP amplitude progressively reduces with age [29]. The only exception was that the ulnar SNAP amplitude was not different between ALS and MMN, probably considering that this sensory nerve was affected in our MMN cohort.

On the other hand, ESC mean values explored with Sudoscan were similar between patients and controls; the age difference between patients and controls and the small number of cases in our cohort may explain this data.

We are aware that our cohort is too small to draw any relevant conclusions, and that is the main limitation of our study. Nevertheless, MMN being a rare disease, it is difficult to collect data from a larger population in a single centre.

A second limitation of our study is the ability of Sudoscan to investigate only autonomic small fibres. However, generally, in neuromuscular disorders, an impairment of autonomic small fibres is always associated with an involvement of somatic small fibres too, and the value of Sudoscan in this setting has been often proved [30,31,32,33].

A third limitation is the lack of different diagnostic tools to assess small fibre neuropathy; we certainly know that a second test to definitively confirm somatic small fibres involvement, such as skin biopsy or laser evoked potentials, or to definitively confirm autonomic small fibres involvement, such as an iodine sweat test, could better clarify this issue, but, unfortunately, patients from our cohort firmly refused to undergo further examinations.

## 5. Conclusions

In conclusion, our results confirm that sensory involvement may be found in MMN, especially after a long disease course. Sensory involvement in this setting is not confined to large fibres, but it can also involve the small fibres. Further studies on a larger population and with different tools are needed to confirm our findings.

## Figures and Tables

**Table 1:** Main demographic, clinical and neurophysiological characteristics of multifocal motor neuropathy, amyotrophic lateral sclerosis, and chronic inflammatory demyelinating neuropathy patients.

Patient, Disease, Gender	Age at Diagnosis	Age at Onset	Age at Examination	Median CMAP (ABP Strength) (R/L)	Median SNAP (R/L)	Ulnar CMAP (ADM Strength) (R/L)	Ulnar SNAP (R/L)	Peroneal CMAP (TA Strength) (R/L)	Peroneal SNAP (R/L)	Sensory Symptoms	Sudoscan Upper Limbs (R/L)	Sudoscan Lower Limbs (R/L)
#1, MMN, M	31	30	34	6.4/*4.7* (5/4)	24.3/22.1	9.1/8.3 (5/5)	12.3/10.7	7.1 (5) (R)	10.4 (R)	no	91/92	87/83
#2, MMN, M	22	20	29	6.8/7.2 (5/5)	31.4/29.3	*2.3*/*4.5* (2/4)	13.3/14.5	5.8 (5) (L)	15.6 (L)	no	88/88	89/87
#3, MMN, M	47	45	54	5.9/*3.4* (5/3)	27.9/24.0	10.1/9.6 (5/5)	10.5/11.3	5.7 (5) (R)	12.3 (R)	no	76/75	87/89
#4, MMN, F	15	14	20	*4.4*/*3.4* (4/3)	42.7/46.7	*3.7*/*2.9* (3/2)	*2.9*/*1.8*	*3.9* (5) (R)	21.7 (R)	yes	*64*/69	85/79
#5, MMN, M	40	39	43	5.3/8.5 (4/5)	7.5/11.8	6.8/7.3 (5/5)	10.6/12.0	5.3 (5) (L)	13.9 (L)	no	*68*/*69*	73/75
#6, MMN, F	41	39	43	9.2/9.3 (5/5)	30.6/29.7	5.4/6.8 (4/5)	7.0/11.3	6.8 (5) (L)	11.8 (L)	no	74/73	76/72
#7, MMN, M	36	35	38	*0.6*/*2.3* (1/3)	13.2/12.4	*0.5*/*2.8* (1/2)	*2.9*/*3.5*	5.0 (5) (L)	7.6 (L)	no	*61*/*62*	73/75
#1, ALS, F	62	61	64	*2.4*/*2.7* (3/3)	14.5/12.7	*2.1*/*2.3* (3/3)	9.3/8.7	*3.1* (4) (L)	7.1 (L)	no	73/74	75/76
#2, ALS, F	67	66	68	*3.2*/*3.5* (4/4)	11.2/9.4	*3.1*/*3.0* (4/4)	7.2/8.7	*3.8* (4) (L)	6.6 (L)	no	72/75	77/78
#3, ALS, F	83	83	84	*2.9*/*2.4* (3/3)	7.9/8.0	*2.1*/*2.6* (3/3)	6.5/7.3	*0.0* (0) (R)	9.1 (R)	no	78/77	80/81
#4, ALS, M	54	53	55	*0.0*/*0.0* (0/0)	12.7/14.3	*0.0*/*0.0* (0/0)	8.9/9.8	*3.5* (4) (R)	11.7 (R)	no	82/80	80/79
#5, ALS, M	63	62	65	*2.3*/*2.1* (3/3)	10.3/10.8	*3.8*/*4.0* (4/4)	6.6/7.0	*0.3* (1) (L)	8.9 (L)	no	82/84	83/85
#6, ALS, M	69	67	70	*3.2*/*1.2* (3/2)	9.4/8.8	*2.8*/*3.0* (3/3)	8.6/9.0	*1.3* (2) (R)	7.9 (R)	no	80/81	78/79
#7, ALS, M	72	70	73	*2.7*/*2.9* (3/3)	11.2/12.7	*2.8*/*3.0* (3/3)	7.9/8.0	*2.3* (2) (L)	8.5 (L)	no	79/80	81/82
#1 CIDP, F	62	60	65	*4.1*/*3.9* (4/4)	*2.3*/*2.7*	*3.7*/*4.0* (4/4)	*1.3*/*2.0*	6.5 (5) (R)	15.0 (R)	yes	*39*/*40*	80/80
#2 CIDP, M	38	38	49	*3.7*/6.8 (4/5)	5.7/*0.9*	11.0/9.2 (5/5)	*3.7*/*1.0*	5.2 (5) (R)	*absent* (L)	yes	80/82	82/84
#3 CIDP, M	68	68	73	7.2/7.5 (5/5)	7.8/8.9	9.3/10.2 (5/5)	6.5/7.2	5.8 (5) (R)	9.5 (R)	yes	73/75	78/80
#4 CIDP, F	57	55	60	*4.1*/*4.0* (3/3)	*absent*/*absent*	*3.5*/*3.8* (3/3)	*absent*/*absent*	*0.7* (2) (L)	*absent* (L)	yes	*56*/*60*	*62*/*61*

Legend of the table: MMN, motor multifocal neuropathy; ALS, amyotrophic lateral sclerosis; CIDP, chronic inflammatory demyelinating neuropathy; M, male; F, female; CMAP, compound muscle action potential; ABP, abductor pollicis brevis; R, right; L, left; SNAP, sensory nerve action potential; ADM, abductor digiti minimi; TA, tibialis anterior; NE, not examined. CMAP amplitude is expressed in mV; SNAP amplitude is expressed in μV; strength is expressed using MRC scale; electrochemical skin conductance of Sudoscan is expressed in μS. Abnormal or borderline values are in *italics*.

**Table 2:** Statistical comparison between MMN patients and control groups.

	Male/Female Ratio	Age at Onset (Mean/Median/SD/Range)	Median CMAP (Mean/Median/SD/Range)	Median SNAP (Mean/Median/SD/Range)	Ulnar CMAP (Mean/Median/SD/Range)	Ulnar SNAP (Mean/Median/SD/Range)	Peroneal CMAP (Mean/Median/SD/Range)	Peroneal SNAP (Mean/Median/SD/Range)	Sudoscan Upper Limbs (Mean/Median/SD/Range)	Sudoscan Lower Limbs (Mean/Median/SD/Range)
MMN	5/2	31.7/35.0/11.2/14–45	Right: 5.5/5.9/2.4/0.6–9.2 Left: 5.5/4.7/2.6/2.3–9.3	Right: 25.4/27.9/10.9/7.5–42.7 Left: 25.1/24.0/11.0/11.8–46.7	Right: 5.4/5.4/3.3/0.5–10.1 Left: 6.0/6.8/2.5/2.8–9.6	Right: 8.5/10.5/4.0/2.9–9.6 Left: 9.3/11.3/4.4/3.5–14.5	5.7/5.7/1.0/3.9–7.1	13.3/12.3/4.1/7.6–21.7	Right: 74.6/74.0/10.6/61–91 Left: 75.4/73.0/10.0/62–92	Right: 81.4/85.0/6.6/73–89 Left: 80.0/79.0/6.0/72–89
CIDP	2/2	55.3/57.5/12.7/49–73	Right: 4.8/4.1/1.4/3.7–7.2 Left: 5.6/5.4/1.6/3.9–7.5	Right: 4.0/4.0/3.0/0–7.8 Left: 3.1/1.8/3.5/0–8.9	Right: 6.9/6.5/3.3/3.5–11 Left: 6.8/6.6/2.9/3.8–10.2	Right: 2.9/2.5/2.5/0–6.5 Left: 2.6/1.5/2.8/0–7.2	4.6/5.5/2.3/0.7–6.5	6.1/4.8/6.4/0–15	Right: 62.0/64.5/15.9/39–80 Left: 64.3/67/5/16.1/40–82	Right: 75.5/79.0/7.9/62–82 Left: 76.3/80.0/9.0/61–84
ALS	4/3	66.0/66.0/9.3/53–83	Right: 2.4/2.7/1.0/0–3.2 Left: 3.3/3.2/1.6/0–7.2	Right: 11/11.2/2.0/7.9–14.5 Left: 11/10.8/2.2/8–14.3	Right: 2.4/2.8/1.1/0–3.8 Left: 2.6/3.0/1.2/0–4	Right: 7.9/7.9/1.0/6.5–9.3 Left: 8.4/8.7/0.9/7–9.8	2.0/2.3/1.4/0–3.8	8.5/8.5/1.5/6.6–11.7	Right: 78.0/79.0/3.7/72–82 Left: 78.7/80.0/3.3/74–84	Right: 79.1/80.0/2.5/75–83 Left: 80.0/79.0/2.7/76–85
MMN vs. CIDP *p* value	0.5758	*0.0106*	Right: 0.611 Left: 0.9466	Right: *0.0044* Left: *0.0041*	Right: 0.4868 Left: 0.6403	Right: *0.0337* Left: *0.0239*	0.2894	*0.0467*	Right: 0.1455 Left: 0.1861	Right: 0.2152 Left: 0.4298
MMN vs. ALS *p* value	1	<*0.0001*	Right: *0.0083* Left: *0.0078*	Right: *0.0049* Left: *0.006*	Right: *0.0415* Left: *0.0017*	Right: 0.707 Left: 0.6056	*0.0001*	*0.0116*	Right: 0.4386 Left: 0.4232	Right: 0.4055 Left: 0.3833

Legend of the table: MMN, motor multifocal neuropathy; ALS, amyotrophic lateral sclerosis; CIDP, chronic inflammatory demyelinating neuropathy; SD, standard deviation; CMAP, compound muscle action potential; SNAP, sensory nerve action potential. CMAP amplitude is expressed in mV; SNAP amplitude is expressed in μV; electrochemical skin conductance of Sudoscan is expressed in μS. Abnormal *p* values are in *italics*.
